# Measurement of intracellular calcium of submandibular glands using a high throughput plate reader

**DOI:** 10.14440/jbm.2017.180

**Published:** 2017-07-17

**Authors:** Abeer Kamal Shaalan, Guy Carpenter, Gordon Proctor

**Affiliations:** Mucosal and Salivary Biology Division, Dental Institute, King’s College London, Guy’s Hospital, Floor 17, Tower Wing, London SE1 9 RT, UK

**Keywords:** calcium, carbachol, *in vitro*, FlexStation, ionomycin, salivary glands, signaling

## Abstract

Calcium ions (Ca^2+^) impact nearly every aspect of cellular life and intracellular calcium [Ca^2+^]_i_ is a critical factor in the regulation of a plethora of physiological functions, including: muscle contraction, saliva secretion, metabolism, gene expression, cell survival and death. By measuring the changes of [Ca^2+^]_i_ levels, critical physiologic functions can be characterized and aberrant pathologic conditions or drug responses can be efficiently monitored. We developed a protocol for assessment of Ca^2+^ signaling in the acinar units of submandibular glands isolated from C57BL/6 mice, using benchtop, multi-mode, high throughput plate reader (FlexStation 3). This method represents a powerful tool for unlimited *in vitro* studies to monitor changes in receptor-mediated Ca^2+^ responses while retaining functional and morphological features of a native setting.

## BACKGROUND

### Key events in salivary gland calcium signaling

**Figure 1.**
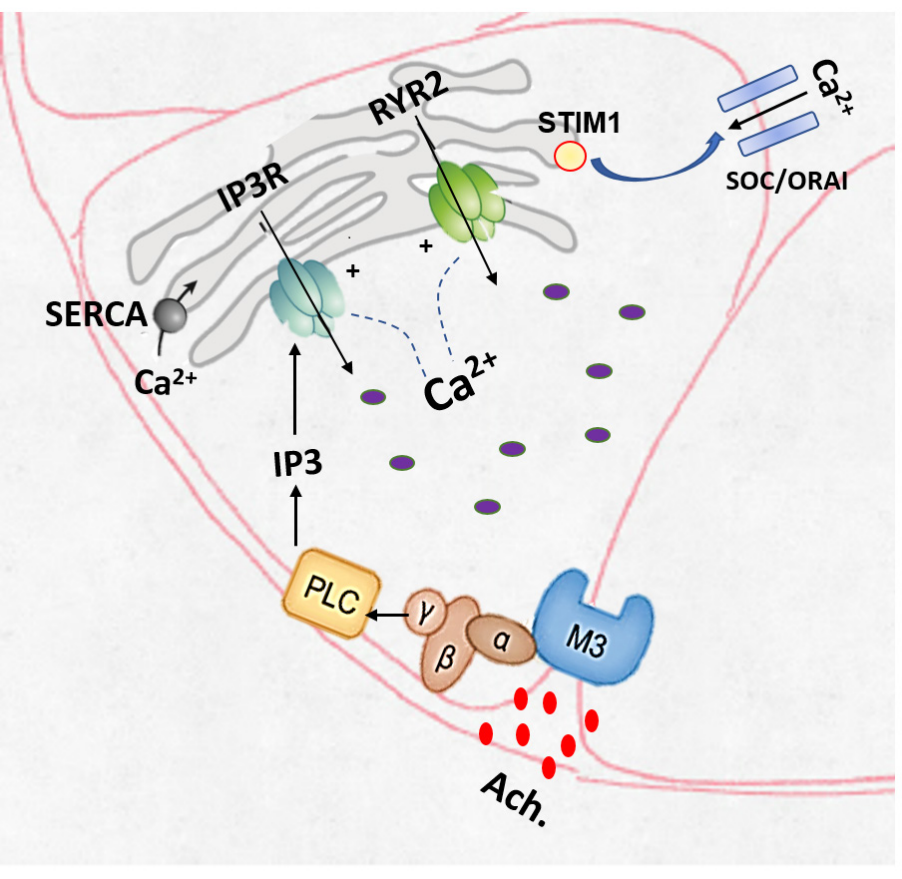
Mechanisms of M3R-mediated release of Ca^2+^ from the endoplasmic reticulum.

For many years, exocrine acinar cells have been the optimal model for examining Ca^2+^ signaling. Acetylcholine is the parasympathetic postganglionic transmitter that mainly acts on the acinar M3 muscarinic receptors to increase intracellular calcium [Ca^2+^]_i_ and stimulate ﬂuid secretion [1]. The primary action of acetylcholine on muscarinic receptors is the activation of phospholipase Cβ (PLCβ). PLCβ cleaves phosphatidylinositol 1,4, bisphosphate (PIP2) to produce diacylglycerol (DAG) and the soluble signaling molecule; inositol 1,4,5, trisphosphate (IP3) [2,3]. IP3 activates the endoplasmic reticulum (ER)-Ca^2+^ release channels; IP3 receptors (IP3Rs), which induce Ca^2+^ release from the ER stores [4]. IP3-mediated Ca^2+^ release *via* IP3R, and resulting depletion of ER Ca^2+^, is the main triggering event in the activation of Ca^2+^ entry, which is mediated by the store-operated Ca^2+^ entry mechanism [5]. It is worth noting that the presence of Ca^2+^ in the cytoplasm will release more Ca^2+^ from the endoplasmic reticulum, providing what’s known as Ca^2+^-induced Ca^2+^ release (CICR), which represents a powerful mechanism for amplification and propagation of cytosolic Ca^2+^ signals [6]. The termination of Ca^2+^ signals is as important as their initiation [7]. Thus, all Ca^2+^ released from the ER is pumped out of the cell by the plasma membrane Ca^2+^ pump (plasma-membrane-Ca^2+^-activated-ATPase, or PMCA) [8]. Extruded Ca^2+^ then re-enters the cell, presumably through store-operated Ca^2+^ channels, to be taken up into the ER by the sarco-endoplasmic-reticulum-Ca^2+^-activated ATPase, or SERCA [9,10] (**Fig. 1**).

### Fura-2 as a calcium sensing dye

**Figure 2.**
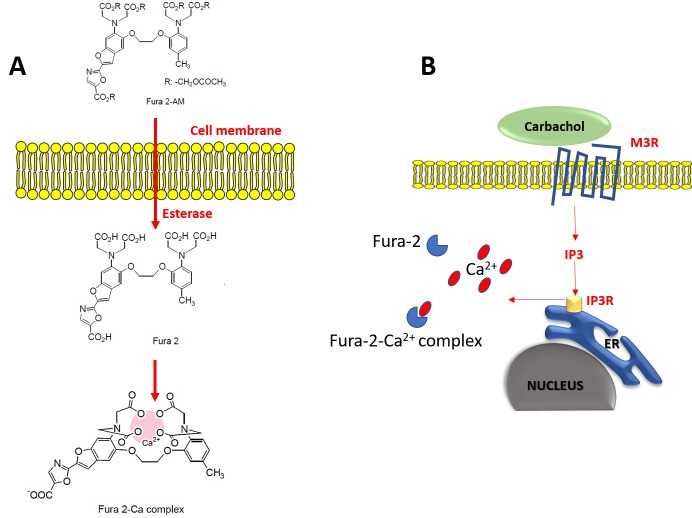
FURA-2 detection of intracellular changes and Ca^2+^ binding. **A.** Structural changes of Fura-2 by esterase activity and Ca^2+^ binding. Fura-2 AM ester is Ca^2+^ insensitive and nonpolar. Once inside the cell, esterase enzymes sequentially cleave the AM groups to leave Fura-2-free acid (Ca^2+^ sensitive, polar) trapped inside the cell, where it is able to bind Ca^2+^. **B.** Carbachol is a muscarinic receptor agonist that initiates a signaling pathway, resulting in the release of Ca^2+^ within seconds. Fura-2 exhibits a calcium dependent excitation spectral shift to report the 340/380 ratio.

Intracellular calcium concentration variations at the nanomolar scale can be detected in live cells with either dyes or biosensors. Fura-2 is by far the most commonly used ratiometric fluorescent dye for quantitative [Ca^2+^]_i_ measurements [11]. Fura-2-free acid is Ca^2+^ sensitive but membrane impermeant. Accordingly, fura-2 pentaacetoxymethyl (AM) ester is used to permeate the cells. Once inside the cell, esterase enzymes sequentially cleave the AM groups to leave fura-2-free acid trapped inside the cell, where it is able to bind Ca^2+^ [12] (**Fig. 2**). Fura-2 exhibits excitation spectrum changes upon Ca^2+^ binding such that the Ca^2+^ free form is excited maximally at 380 nm while the Ca^2+^ bound form is excited maximally at 340 nm. Both forms emit fluorescence with a peak at 510 nm [13]. Therefore, it follows that the concentration of free [Ca^2+^]_i_ is proportional to the ratio of fluorescence at 340/380.

### High throughput assay of calcium signaling

Multi-mode, automated, microplate readers with integrated fluidics transfer capabilities can measure time-resolved changes in fluorescence and are suitable for performing intracellular Ca^2+^ mobilization assays in a 96-well format. The FlexStation 3 has been used for studying the function of Ca^2+^-permeable ion channels and G-protein coupled receptors in the HTC4 rat hepatoma cell line [14]. In addition, it has permitted monitoring the ryanodine receptor 2 (RYR2) activity in the mouse pulmonary artery myocytes [15] and P2 receptors’ (P2Rs) activity in eosinophils isolated from rat peritoneal lavage [16]. To our knowledge, the FlexStation 3 has not been used to study calcium kinetics in cells prepared from exocrine tissues such as the salivary glands. While measuring [Ca^2+^]_i_ using single cell fluorescent microscopy provides sensitivity and temporal resolution, and it is also exhaustive and time consuming.

Accordingly, the high-throughput screening of [Ca^2+^]_i_ kinetics with the FlexStation 3 can be more favorable, mainly because of its cost-effectiveness, speed and the automated fluidics system. Furthermore, the use of this instrument for measurements of intracellular Ca^2+^ in salivary gland cells or any acutely isolated cells from other tissues provides two overlapping advantages. First, the possibility of automated screening of multiple wells at the same time, overcomes the delay that can be anticipated with linear systems measuring one well at a time. Reasonably, for real-time measurements of several minutes, linear systems introduce an undesirably long delay between measurement of the first and last well and the kinetic Ca^2+^ measurements of only a few minutes/well will introduce a delay of several hours between the first and the last measurement in a 96-well plate. During this delay, cells maintained in suboptimal conditions, may show deterioration of function and viability. In addition, this could result in dye leakage as well as dye sequestration in intracellular organelles, *e.g.*, mitochondria [17]. The other advantage that can also be related to the former fact is the possibility of automatic and simultaneous transfer of a wide array of compound concentrations sequentially to the assay plate. This allows repeatable, reliable and reproducible recording of Ca^2+^ levels in the acinar units, which can be differentially treated. The step-by-step protocol presented herein describes a method that has been developed for preparing mouse salivary glands for assessing acinar cell Ca^2+^ dynamics *ex-vivo*. This approach leverages multi-well plate assaying of calcium signals with improved workflow in a semi-automated, high volume fashion. Presumably, this protocol can be applied to other exocrine glands, to reliably unravel aberrations in [Ca^2+^]_i_ responses, which can directly cause cell dysfunction and increase the rates of cell damage [18].

## MATERIALS

### Reagents

- HEPES (Sigma-Aldrich, Cat. # H3375)
- NaCl (Sigma-Aldrich, Cat. # S7653)
- KCl (BDH, AnalaR, Cat. # 101984L)
- MgCl_2_ (Sigma-Aldrich, Cat. # M8266)
- CaCl_2_ (Sigma-Aldrich, Cat. # C-4901)
- Glucose (Sigma-Aldrich, Cat. # G8270)
- Glutamine (Sigma-Aldrich, Cat. # G7513)
- MEM Non-Essential Amino Acids Solution (100X) (Thermo Fisher Scientific (Life Technologies) Cat. # 11140050)
- Bovine serum albumin (BSA) (Sigma-Aldrich, Cat. # A2153)
- Collagenase from Clostridium histolyticum (Type-4 collagenase) (Sigma-Aldrich, Cat. # C5138)
- Soybean Trypsin Inhibitor (Thermo Fisher Scientific (Life Technologies), Cat. # 17075029)
- Corning^®^ Cell-Tak (Fisher Scientific Ltd, Cat. # 354240)
- NaHCO_3_ (Sigma-Aldrich, Cat. # S6014)
- Fura-2 AM (Molecular Probes™, Cat. # F-1201)
- Probenecid (Sigma-Aldrich, Cat. # P8761)
- Half-area, 96-well plates ((Fisher Scientific Ltd, Cat. # 10717804)
- Carbachol (CCh) (Santa Cruz Biotechnology, Cat. # sc-202092)
- Ionomycin (IM) (Santa Cruz Biotechnology, Cat. # sc-3592)
- Culture grade dimethyl sulfoxide (DMSO) (Sigma-Aldrich, Cat. # 276855)
- Dulbecco phosphate buffered saline (DPBS) (Sigma-Aldrich, Cat. # D8662)

### Recipes

- HEPES incubation buffer: 20 mM HEPES, 95 mM NaCl, 4.7 mM KCl, 0.6 mM MgCl_2_, 1.3 mM CaCl_2_, 10 mM glucose, 2 mM glutamine, and 1 × minimum Eagle’s medium non-essential amino acids, pH 7.4. The buffer was oxygenated for 20 min before use
- BSA incubation buffer: BSA 1% w/v final added to 25 ml of the HEPES buffer
- Collagenase digestion buffer (CDB): 1.1 mg/ml type-4 collagenase and 1 mg/ml soybean trypsin inhibitor added to 6 ml of BSA incubation buffer
- Sodium bicarbonate (NaHCO_3_) neutral buffer solution: 0.1 M sodium bicarbonate, pH 8.0 was prepared by dissolving 420 mg NaHCO_3_ in 50 ml ultrapure distilled water
- For coating of a half area 96-well plate: Dilute 30 µl of Corning^®^ Cell-Tak in 2 ml of the neutral bicarbonate buffer
- Fura-2 AM stock solution: Suspend 1 mg of lyophilized Fura-2 AM with DMSO to yield a 1 mM stock. Aliquot this stock and keep at all times in the dark at **−**20°C
- 1 M probenecid: Dissolve in 1 M NaOH (50 mg/ml), yielding a clear, colorless solution
- Fura-2 working solution: 4 µl Fura-2 AM stock, 4 µl probenecid 1 M and 4 ml HEPES buffer

**CAUTION:** Buffer preparation and cell loading should be performed in the dark to prevent degradation of the Fura-2.

### Equipment

FlexStation 3 (Molecular Devices, Inc.) benchtop scanning fluorometer is used to measure changes in fluorescence of the fura-2 stained acinar units upon agonists’ transfer from the compound plate to the pre-designated set of wells in the assay plate.

## PROCEDURE

**Figure 3.**
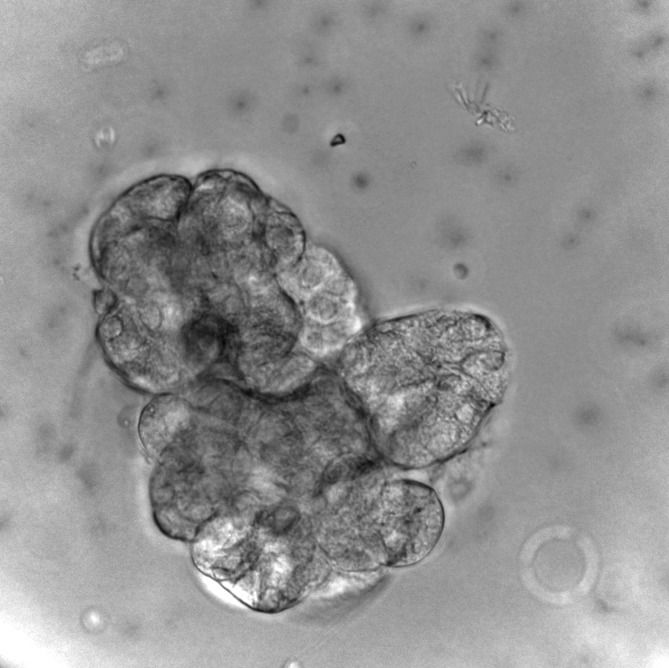
Representative example of the physiologic acinar units obtained in the present protocol following collagenase digestion.

**Figure 4.**
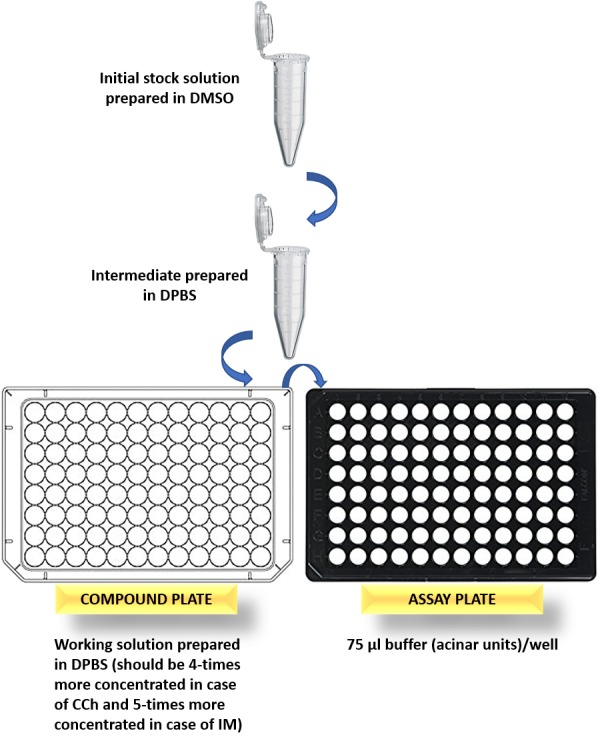
Step-wise illustration of compound plate preparation.

1. Assay plate preparation
  To maintain acinar units in place throughout the FlexStation 3 measurements, Corning^®^ Cell-Tak adhesive was used to coat the half-area, 96-well assay plates which were used in these experiments. Corning^®^ Cell-Tak adhesive is a formulation of the “polyphenolic proteins” [19] extracted from the marine mussel, Mytilus edulis. This family of related proteins is the key component of the glue secreted by the mussel to anchor itself to solid structures in its natural environment [20].
  **1.1.** On the day preceding the experiment, filter-sterilize the NaHCO_3_ neutral buffer solution.
  **1.2.** The amount of Corning^®^ Cell-Tak required for each well in the assay plate is calculated according to the manufacturer’s recommendations: 0.56 µg Corning^®^ Cell-Tak/well.
  **1.3.** Dilute the correct amount of Corning^®^ Cell-Tak into the neutral buffer, mix thoroughly, and dispense into the assay plate wells within 10 min.
  **1.4.** Place the cover and incubate the coated assay plate overnight at room temperature.
  **1.5.** On the next day (the day of the experiment), pour the unevaporated Cell-Tak off and wash each well with 200 µl filter-sterile distilled water to remove the bicarbonate.
    **HINT:** It is of extreme importance not to place the Cell-Tak-coated assay plate in the CO_2_ incubator while preparing the compound plate, otherwise Cell-Tak will lose its activity and acinar units will be detached from the plate bottom when the secretagogues are added and severe inconsistency in fluorescence recording will occur.
2. Isolation and preparation of the SMGs
  **2.1.** Dissect the submandibular glands (SMGs) and rinse it with Hanks balanced salt solution.
  **2.2.** Mince the excised SMG with scalpels or curved scissors in a labeled weighing boat, containing 1 ml of the CDB.
  **2.3.** Transfer the gland homogenate to a 50 ml falcon tube and incubate in 4 ml CDB, in a 37°C water bath for 30 min.
  **2.4.** After the digestion is complete, carefully pipette-out the CDB and replace with 6 ml of BSA incubation buffer.
  **2.5.** Shake the tube vigorously by hand for 10 s, in order to disperse the cells into smaller acinar units (**Fig. 3**).
  **2.6.** Allow the acinar units to settle, then discard the supernatant and replace with HEPES buffer-containing Fura-2 AM.
    **HINT:** Care should be taken to remove the BSA buffer completely, followed by replacement with HEPES incubation buffer.
3. Dye loading
  **3.1.** To prevent leakage of the dye from the cells, an organic anion transport blocker probenecid was added to the dye buffer to achieve a final concentration of 1 mM.
  **3.2.** Incubate the acinar units (in the falcon tube) in 4 ml Fura-2 working solution in a CO_2_ incubator at 37°C for 1 h.
  **3.3.** During this period, switch on the FlexStation and adjust the temperature to 37°C.
  **3.4.** After one hour, wash the acinar units with HEPES buffer once.
  **3.5.** Calculate the final HEPES buffer volume to be dispensed on the acinar units according to number of wells to be seeded, using the following formula: final HEPES buffer per gland = number of wells to be seeded × 75 (final volume/well in the assay plate).
  **3.6.** After seeding the HEPES buffer/acinar units into the assay plate, cover it and place it into its allocated position in the FlexStation, until preparation of the compound plate is complete.
    **CAUTION:** Do NOT do this step in the CO_2_ incubator.
    **HINT:** (1) It is essential to take into consideration the importance of having relatively equal density of acinar units per well. To achieve pipetting the digested acinar units gently up and down frequently between each transfer of HEPES buffer/acinar units into the wells of the assay plate, we recommend performing this critical step near an inverted microscope to frequently check uniform density of the seeded acinar units; (2) Since probenecid also has effect on some ion channels, caution should be taken when interpreting data obtained from experiments that include probenecid in the assay buffer.
4. Compound plate preparation
  **4.1.** Prepare the stock solutions of the cholinergic receptor agonist; carbachol (CCh) and the calcium ionophore; ionomycin (IM) in DMSO.
  **4.2.** Prepare the intermediate and working solutions in DPBS (**Table 1**).
    **CAUTION:** (1) Since the FlexStation will be set-up to transfer 25 µl CCh followed by 25 µl IM, from the compound plate columns to the 75 µl buffer/acinar units’ columns in the assay plate. Accordingly, it is very important to take into consideration that determination of the final concentrations in the compound plate, depends on the buffer volume in the corresponding well of the assay plate, before the compound addition (**Fig. 4**); (2) It is very important to note that the compound concentration is subject to change after each compound addition (in experiments where multiple compounds are used).
5. FlexStation settings
  **5.1.** Set-up the FlexStation 3 to record changes in calcium signals before (baseline) and after compound additions, as demonstrated in **Table 2**.
  **5.2.** For baseline fluorescence reading, adjust the settings similar to that demonstrated in **Table 2**, except that: (1) select an endpoint read type; (2) do not select the compound transfer option.
  **5.3.** Record emission ratios with excitation wavelengths of 340 and 380 nm every 6 s after compound applications, for 3 min.
  **5.4.** Experimental data is processed directly using the SoftMax Pro software (otherwise it can be copied and pasted into any spreadsheet program, such as Microsoft Excel).

## RESULTS

**Figure 5.**
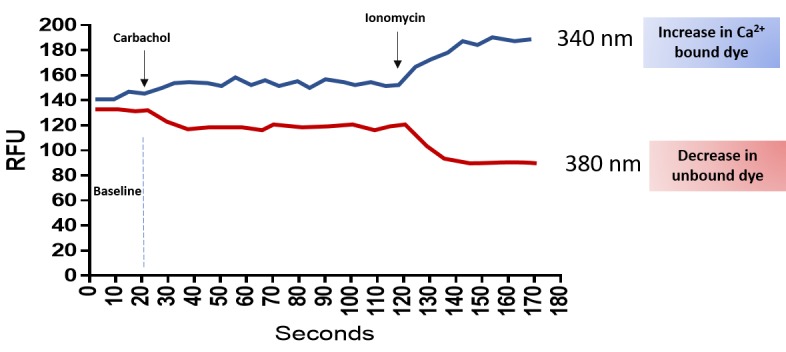
Schematic illustration of intracellular calcium changes as recorded by flexstation at 340 and 380 nm. The raw data depicts the typical signals obtained from a fura-2-loaded cell when it is excited at 340 and 380 nm. Agonist stimulation will cause an increase in the 340 nm signal and a decrease in the 380 nm signal. Addition of an ionophore (Ionomycin) will release Ca^2+^ from all intracellular stores and will result in the F_340max_ and F_380min_.

Carbachol is a cholinergic agonist which evokes intracellular Ca^2+^ signaling by increasing IP_3_ concentrations and releasing Ca^2+^ from the ER stores [22]. On the other hand, ionomycin is known to increase [Ca^2+^]_i_ in virtually all cell types, including salivary acinar cells, *via* globally depleting Ca^2+^ from all intracellular stores [17,23]. Fura-2 ratiometric properties can be depicted in real time through the dual excitation wavelength capability of the FlexStation. **Figure 5** exemplifies the raw graph obtained using the SoftMax Pro software and demonstrates that when Fura-2 is excited at 340 nm, its emission is raised with increasing Ca^2+^ concentration, whereas when excited at 380 nm, Fura-2 emission is decreased with increasing Ca^2+^ concentration. Changes in the 340/380 ratio will reflect changes in intracellular-free Ca^2+^ concentrations.

**Figure 6.**
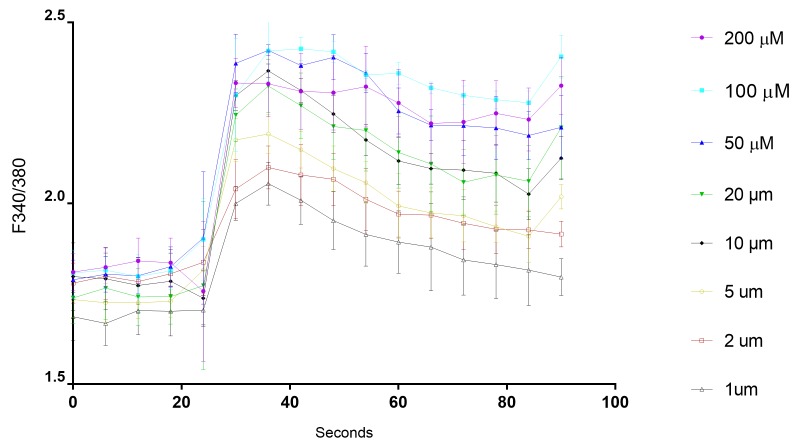
Analysis of calcium mobilization in mouse SMG acinar units. FlexStation 3 was set-up to add CCh after 20 s of baseline measurement. Supraphysiological (200-2 µM) and physiological (1 µM) carbachol concentrations stimulated calcium release from the ER in a dose-dependent manner. Generally, the most prominent acinar responses were perceived with the supraphysiological CCh doses. Data represent the 340/380 ratios recorded for 90 s by FlexStation 3.

In the present experiments, the processed data (F340/380) revealed that CCh induced a dose-dependent increase of [Ca^2+^]_i_ levels. Prompt [Ca^2+^]_i_ mobilization from the acinar ER stores in response to CCh was reflected as a fluorescence signal spike, that peaked for 12 s after the secretagogue application. This peak was then followed by a plateau phase, remarkably higher than the resting [Ca^2+^]_i_, which usually had Ca^2+^ oscillations superimposed on it (**Fig. 6**). The rapid initial increase in [Ca^2+^]_i_ due to the activation of muscarinic receptors by agonists has been shown to be mediated through the release of IP3 and the subsequent mobilization of Ca^2+^ from internal stores [24]. Previous studies have shown that the early Ca^2+^ oscillations following CCh stimulation were originated from the ER, by the interplay of repetitive releases and re-uptake of Ca^2+^ by IP3-dependent Ca^2+^ channels and SERCA pump activity, respectively [7].

**Figure 7.**
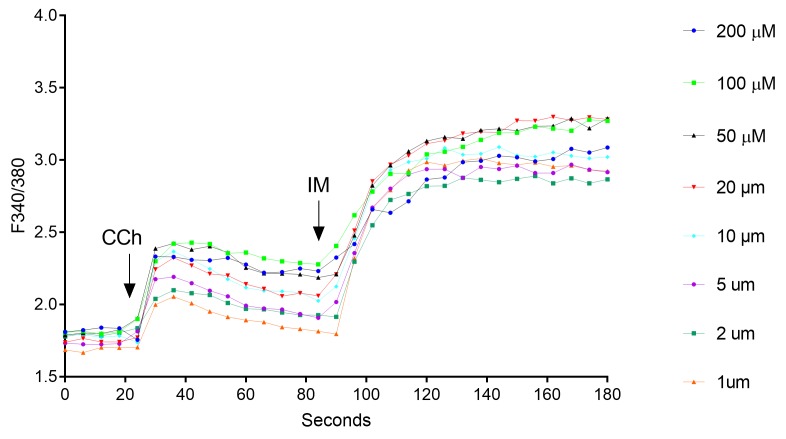
Representative graph showing enhancement of the 340/380 ratios in response to 6 µM ionomycin. FlexStation 3 was set-up to add the IM 70 s after CCh application.

The Ca^2+^ ionophore ionomycin is a pharmacological tool with which to evoke changes in [Ca^2+^]_i_ which bypass the receptor-operated mechanisms [25]. As a positive control, IM (6 µM) was added to the acinar units to demonstrate the global Ca^2+^ release from intracellular stores. As seen in **Figure 7**, addition of IM induced a fast F 340/380 peak followed by a rising plateau phase. Previous studies demonstrated that IM increases submandibular acinar [Ca^2+^]_i_ *via* two pathways in a concentration-dependent manner. One is the Ca^2+^/H^+^ exchange, which is mediated by a high concentration of IM, such as 5 µM. The other is the store-operated Ca^2+^ entry channels (SOCs), which are activated by a low concentration of IM, such as 1 µM [24].

## TROUBLESHOOTING

The present study has shown that a multimode plate reader with integrated fluidics (Flexstation 3) can be used to make multiple and repeated measurements of intracellular Ca^2+^ in order to determine the responsiveness of salivary gland acini to changing concentrations of autonomimetics. This approach allows the study of cells in their native conformation, avoiding the problems associated with single cell dispersions and immortalized cell lines. However, a limitation of acutely isolated cells of this type cannot be used over an extended period of time and experiments should be performed on the day of preparation. Future studies could be undertaken in order to determine the applicability of this protocol to other exocrine glands. Furthermore, changes to the tissue preparation protocol might enable use of the technique to organ cultures of embryonic salivary glands or salivary tissue grown *in vitro* on different scaffolds. **Table 3** provides the most common problems that can encountered during performance of this protocol.

## Figures and Tables

**Table 1.** Carbachol and ionomycin preparation.

	Intermediate	Final conc. (µM) in assay plate	Initial conc. (µM) in compound plate	CCh (µl)	IM (µl)	Buffer (µl)
Carbachol stock solution (100 mM): 100 mg CCh in 5.47 ml DMSO	1:100 in DPBS	50 20 10	200 80 40	40 16 8		160 184 192
Ionomycin stock solution (3 mM): 5 mg IM in 2.23 ml DMSO	No intermediate is required	6	30		2	198

**Table 2.** FlexStation settings.

**Read mode**	FL
**Read type**	Flex
**Category**	
• **Wavelength** [2 wave length pairs]	Excitation: Lm1 340 nm Lm2 380 nm Emission: Lm1 510 Lm2 510 Auto cut-off
• **Plate type**	96-well Corning half area flat clear bottom
• **Read area settings**	Select all (read the whole plate)
• **PMT and optics settings**	PMT gain: Medium Flashes per Read: 6
• **Timing settings**	Total run time: 3 mins Interval: 6 secs Number of reads: 21
• **Compound transfer**	
**Number of transfers**	2
**Initial volume (µl)**	Compound 1 (CCh): 75 Compound 2 (IM): 100
Pipette height*	Compound 1 (CCh): 90 Compound 2 (IM): 100
**Volume**	Compound 1 (CCh): 25 Compound 2 (IM): 25
Rate (µl/sec)*	Compound 1 (CCh): 2 Compound 2 (IM): 2
**Time point**	Compound 1 (CCh): 20 Compound 2 (IM): 120
• **Compound plate type**	Costar 96 opaque 3 ml
• **Pipette tips and layout**	Subject to experimental condition
• **Compound and tips column**	Subject to experimental condition
**No trituration (otherwise dislodgment of the Cell-Tak adherent cells will occur)**	

*The parameters for the integrated FlexStation pipettor require optimization for each assay. The dispensation height of the pipettor and the speed of dispensation should be adjusted to ensure optimal delivery of the compounds to the specific plates being used. Optimal delivery should not cause cell disruption but should allow adequate mixing of the compounds in the well. To assist adequate mixing of compounds, the volume of agonist added to the well is typically 25% of the final well volume. The optimal dispenser speed may vary according to how well cells adhere to the bottom of the well [21].

**Table 3.** Troubleshooting.

Step	Problems	Causes	Suggestions
Assay plate preparation	Cells detached from the assay plate bottom	Incorrect preparation of Corning^®^ Cell-Tak Incubation of the Cell-Tak-coated assay plate in CO_2_ incubator Residual BSA in the incubation buffer	Follow the Corning^®^ Cell-Tak preparation data sheet accurately Never incubate Cell-Tak-coated surfaces in a CO_2_ incubator Wash BSA from the incubation buffer before dispensing the cells in the Cell-Tak-coated assay plate
Isolation and preparation of the SMGs	SMGs yield single cells rather than glandular physiologic units	Over-digestion of the SMGs or using the improper collagenase	Do not increase the digestion time, use the proper collagenase type and concentration
Dye loading	Fura 2 binding to BSA	Residual BSA in the incubation buffer prior to Fura-2 loading over the physiologic units	It's critical that the Ca^2+^ dye is loaded in a BSA-free incubation buffer
